# Uncommon Cause of Meningitis: Lactobacillus mucosae Meningitis in a Patient With Recurrent CSF Leak Post-craniotomy

**DOI:** 10.7759/cureus.97414

**Published:** 2025-11-21

**Authors:** Douglas Asede, Francis Demiraj, Ian Rampasad, Sujai Nath, Roozbeh Nazarian

**Affiliations:** 1 Neurology, Charles E. Schmidt College of Medicine, Florida Atlantic University (FAU), Boca Raton, USA; 2 Internal Medicine, Charles E. Schmidt College of Medicine, Florida Atlantic University (FAU), Boca Raton, USA; 3 Marcus Neurosciences Institute, Boca Raton Regional Hospital, Boca Raton, USA

**Keywords:** all neurology, bactrial meningitis, cranio-facial surgery, endoscopic csf leak repair, spinal csf leak

## Abstract

Meningitis is a serious infection with potentially life-threatening consequences if not promptly treated. Common bacterial pathogens include *Streptococcus pneumoniae*, *Neisseria meningitidis*, and *Haemophilus influenzae*. However, in rare instances, less common organisms can cause meningitis, particularly in individuals with predisposing conditions, such as a cerebrospinal fluid (CSF) leak. Here we present the case of a 63-year-old male with a history of craniotomy and CSF leaks who developed meningitis due to *Lactobacillus mucosae*. He presented with severe headaches, photophobia, diaphoresis, nasal discharge, and nuchal rigidity. He was found to have a CSF leak confirmed by positive beta-2 transferrin. CSF analysis revealed elevated WBCs, elevated protein, and normal glucose. Blood cultures grew *Lactobacillus mucosae*. MRI brain revealed a depression of the cribriform plate with cortical discontinuity and a small encephalocele, likely the source. The patient was successfully treated with a total of 10 days of intravenous antibiotics and steroids and was transferred to another facility for neurosurgical repair. No recurrence was reported 8 months post-treatment during a subsequent hospitalization for an unrelated issue. Lactobacillus meningitis is rare in the literature; to our knowledge, this is the first case involving *Lactobacillus mucosae*. This case illustrates the importance of considering atypical pathogens in patients with risk factors such as cranial surgery and CSF leaks who present with signs of meningitis. The presence of an encephalocele and cortical discontinuity further complicated the clinical picture, requiring a careful and coordinated approach to management.

## Introduction

Meningitis is characterized by inflammation of the membranes that surround the brain and spinal cord. Viral and bacterial diseases are the main causes. *Streptococcus pneumoniae, Neisseria meningitidis, and Haemophilus influenzae* are three common causes [1]. However, in recent years, there has been a growing awareness of uncommon bacterial causes of meningitis in patients with immunosuppression, central nervous system (CNS) abnormalities, or recent neurosurgical interventions [2,3,4]. Patients with a history of head trauma, craniotomies, or recurrent cerebrospinal fluid (CSF) leaks are particularly vulnerable due to compromised protective barriers/membranes around the CNS, which can allow non-pathogenic organisms into the sterile CNS environment [5,6].

CSF leaks arise from defects in the skull base or spinal dura, facilitating communication between the typically sterile CNS and non-sterile areas, including the nasal cavity or middle ear. This breach undermines CNS sterility and establishes a potential entry point for microorganisms, increasing the risk of meningitis in patients. *Lactobacillus* species are generally considered commensal or probiotic organisms found in the gastrointestinal, upper respiratory, and genitourinary tracts.

*Lactobacillus* spp. is well known for its role as a commensal bacterium in the gut and genitourinary tract. However, in situations where anatomical barriers are compromised or immune function is impaired, they may function as opportunistic pathogens. This shift emphasizes the importance of clinicians remaining watchful for unusual organisms, especially in patients with predisposing factors such as head trauma, craniotomies, CSF leaks, or impaired immune systems.

Here, we report a rare case of Lactobacillus meningitis in a patient with a history of craniotomy and recurrent CSF leaks, who presented with severe headache, photophobia, nasal discharge, and nuchal rigidity with leukocytosis in the blood and CSF. Brain imaging revealed encephalocele and cortical discontinuity, which likely created a structural entry point for *Lactobacillus mucosae*, allowing this typically noninvasive bacterium to infect the meninges.

## Case presentation

The patient was a 63-year-old male who presented to our emergency department with right nasal discharge, a headache rated as 10/10, and diaphoresis. His medical history was notable for septoplasty and craniotomy with two dural repairs after a motor vehicle collision as a cyclist, which was complicated by cerebrospinal fluid (CSF) leakage in 2019. The patient had not had any recurrences of CSF leaks after neurosurgical repair but stated that his original episode had presented with right nasal discharge after trauma. He had, however, developed intermittent right nasal discharge a week before admission. The patient denied any fevers at home but did report diaphoresis, photophobia, nausea, and vomiting. Of note, he had received Pneumovax 23 three months before admission. He stated that he might have used probiotics six months before the development of symptoms and had undergone a routine dental visit three months before presentation.

In the emergency department (ED), the patient was hypertensive (BP 167/72 mmHg) and tachycardic (heart rate 101 beats per minute). On evaluation, the patient was diaphoretic and febrile (102.5 °F), with nuchal rigidity and mild bilateral papilledema. Laboratory studies were notable for leukocytosis (WBC 16,700/µL). A chest X-ray revealed no acute cardiopulmonary process. The brain CT was negative for acute intracranial abnormality, and the CT of the internal auditory canals/temporal bones showed small-volume frothy secretion in the paranasal sinuses, likely reflecting active sinusitis. The patient was started on intravenous antibiotics (vancomycin 1.5 g, ceftriaxone 2 g), morphine 4 mg for pain, and dexamethasone 15 mg (0.15 mg/kg) q6h to reduce inflammation and improve outcome [6,7]. The patient was admitted for further evaluation and management of meningitis. Treatment was escalated to vancomycin 1.75 g every 12 hours, ampicillin 2 g every 4 hours, and cefepime 2 g every 8 hours for broader coverage. CSF analysis (Table 1) revealed elevated WBCs (9,873/µL, corrected 9,870/µL), elevated RBCs (2,000/µL), elevated protein (942 mg/dL), and normal glucose (57 mg/dL). Given the polymorphonuclear (PMN) predominance in the CSF findings, our team chose not to initiate acyclovir due to the low probability of viral meningitis.

**Table 1:** CSF analysis revealed elevated protein and WBC counts, with predominant polymorphonuclear cells suggestive of a bacterial infection.

CSF findings	Results	Normal
Gross appearance	Cloudy	Clear
White blood cells	9,873/µL	0-5
Polymorphonuclear	92%	<5
Mononuclear	8%	<5
Red blood cells	2,000/µL	0
Glucose	57 mg/dL	45-75
Protein	942 mg/dL	15-45

**Figure 1:**
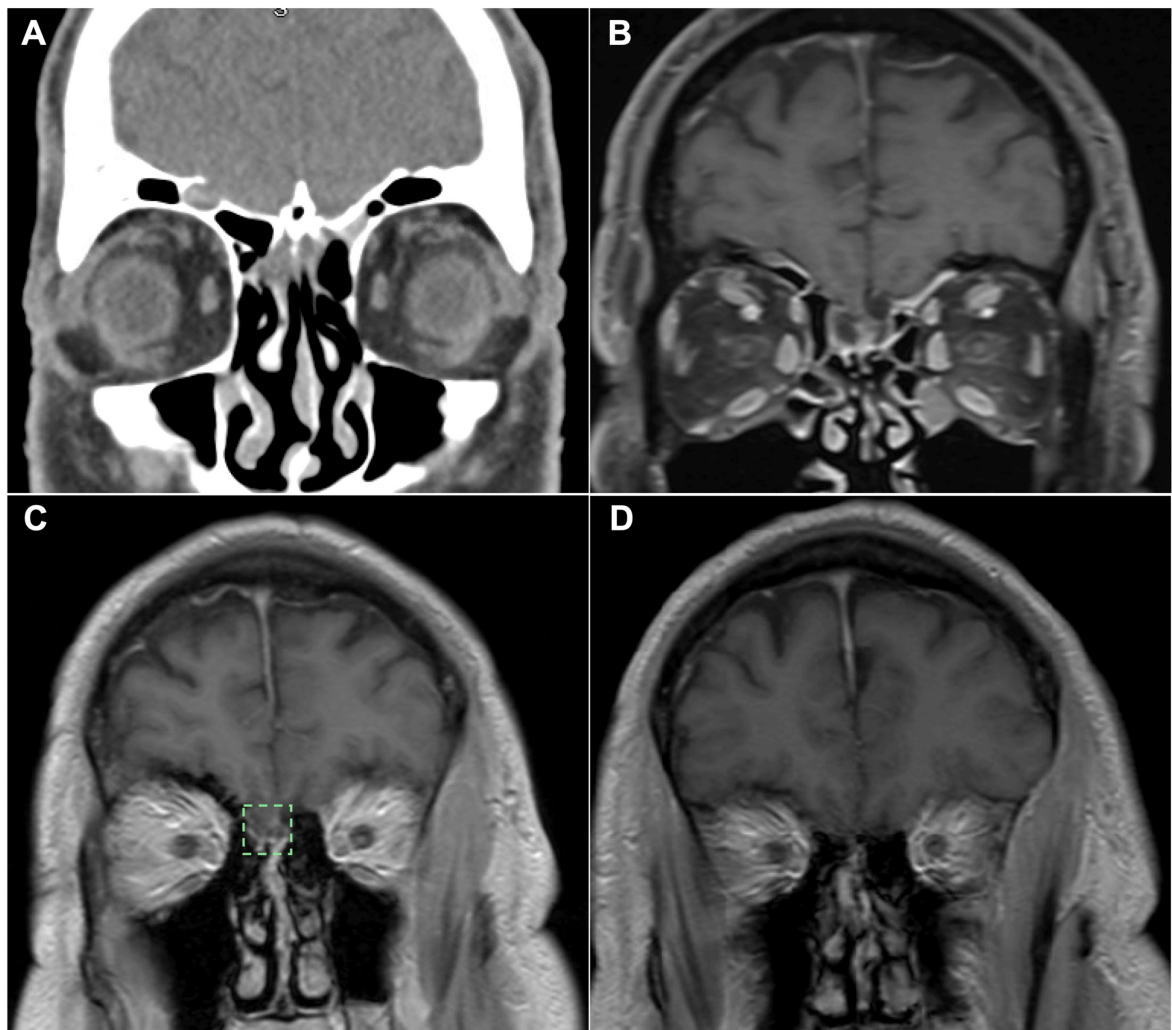
Evidence for a possible source of infection. (A) Coronal CT of the brain showing widened bilateral lamellae of the cribriform plate. (B-D) Coronal T1-weighted post-contrast MRI images. (B) Corresponding coronal MRI section demonstrating an enlarged lamella similar to the CT image in (A). (C) Depression of the cribriform plate with cortical discontinuity and a small amount of brain tissue rather than CSF on the right, suggestive of a small encephalocele (highlighted in green). (D) The posterior frontal sinus table is dehiscent and opacified.

On day 3 of admission, blood cultures (2/2) grew gram-positive rods identified as *Lactobacillus mucosae*, which were sensitive to penicillin. Urine and respiratory cultures were negative. Therefore, cefepime was discontinued, and treatment was de-escalated to vancomycin 1.75 g every 12 hours and ampicillin 2 g every 4 hours only. MRI of the brain revealed a depression of the cribriform plate with cortical discontinuity and a small encephalocele (Figure 1), which likely served as the source of the infection. Nasal discharge was collected and tested positive for beta-2 transferrin, a highly specific marker for CSF. The patient continued ampicillin and vancomycin and completed a total of 10 days of intravenous antibiotics. He experienced significant clinical improvement with therapy and remained afebrile throughout his hospitalization. Papilledema and nuchal rigidity resolved by day 3 of hospitalization, and he had returned to his baseline mentation. He completed a standard four-day regimen of dexamethasone at 15 mg every 6 hours, followed by an additional dose of 15 mg every 12 hours because of an intermittent mild (2/10) headache, which subsequently resolved on day 5 of admission. Our institution lacked skull base surgical subspecialists, and he would have to be transferred to a tertiary care center for open skull base surgical repair. The patient was ultimately transferred to a tertiary center, where he underwent dural repair and shunt placement, resulting in a consistent recovery and a significant improvement in symptoms.

## Discussion

*Lactobacillus* spp. are non-pathogenic, commensal organisms in the gastrointestinal and genitourinary tracts; however, they are not a common cause of meningitis. We report for the first time a case caused by the *Lactobacillus mucosae* species.

This patient’s history of head trauma, craniotomy, and confirmed recurrent CSF leaks poses a significant risk for the introduction of pathogens into the CNS. Additionally, imaging revealed the underlying anatomical defect, with a depression of the cribriform plate and a small encephalocele, likely providing direct access for bacteria to enter the CNS. Although the CSF culture was negative (likely reflecting prior antibiotic exposure), blood cultures were positive for *Lactobacillus mucosae.* This discordance-sterile CSF with positive blood cultures has been well documented and can occur when antibiotics are initiated before lumbar puncture (LP) and CSF sample collection [8], consistent with guidelines recommending immediate antimicrobial therapy while awaiting additional diagnostic studies in cases with a high suspicion of bacterial meningitis [7,9]. This finding is clinically significant: prior antibiotic therapy reduces CSF culture yield, so a negative CSF culture does not rule out bacterial meningitis. Multiple guidelines and observational studies highlight that blood cultures are important diagnostic adjuncts when CSF cultures are negative or when LP is delayed. In practice, blood culture positivity can establish the etiologic diagnosis and guide targeted therapy even when CSF is sterile [9,10,11].

Although Lactobacillus infection is often associated with immunocompromised states or disruption of mucosal barriers, the concurrent positive blood cultures, a compromised CNS barrier, compatible clinical features, and CSF profile implicate *Lactobacillus mucosae* as the rare cause of meningitis in this study.

Treatment of anaerobic bacterial meningitis, including Lactobacillus meningitis, remains a challenge due to the organism’s nature and variable susceptibility to antibiotics [11]. In this case, the patient was successfully treated with combination antibiotic therapy for optimum coverage. In addition, dexamethasone was administered to address the inflammatory response caused by the infection and enhance the overall outcome. This treatment strategy aligns with standard guidelines for the management of bacterial meningitis, which emphasize the significance of early antibiotic therapy and supportive care.

The etiology of CSF leaks is traumatic in over 80% of cases [12]. One key element of this case was that this was a recurrent CSF leak in a patient with a history of craniotomy. In a cohort of 1,083 CSF that underwent repair, 10.3% would have a recurrence, and a majority of these were in the anterior skull base, as seen in our case [13]. The overwhelming majority of these reoccurrences were within 12 months of initial repair, which creates a contrast to our patient's leak that occurred over 60 months from his initial repair. Furthermore, *Lactobacillus* spp. have been reported in the nasal mucosa in healthy people and disease states [14]. Possible mechanisms for CSF leak reoccurrence include breakdown of the dura mater tissue due to microtrauma or scar atrophy, given the presence of depression on the cribriform plate and an encephalocele [12,15]. Ultimately, the patient was transferred to a different facility for neurosurgical repair of the encephalocele to prevent the recurrence of the CSF leak and subsequent episodes of meningitis [16]. In the event of recurrent CSF breaches, the surgical correction of the defect is essential, as it prevents direct communication between the sterile CNS environment and the external environment, thereby reducing the likelihood of future infections. The patient was seen eight months post-treatment during a subsequent hospitalization for an unrelated condition, and they reported no recurrence.

## Conclusions

This case emphasizes the significance of a high index of suspicion, the consideration of rare pathogens in patients with predisposing factors, and the need for prompt intervention to ensure the best possible patient outcome. It also emphasizes the necessity of a multidisciplinary approach that encompasses both medical and surgical interventions to achieve a favorable outcome in situations that are as intricate as this.
